# Rare Mutations in AHDC1 in Patients with Obstructive Sleep Apnea

**DOI:** 10.1155/2019/5907361

**Published:** 2019-10-13

**Authors:** Song Yang, Kun Li, Miao-Miao Zhu, Xian-Dao Yuan, Xiao-Lu Jiao, Yun-Yun Yang, Juan Li, Linyi Li, Hui-Na Zhang, Yun-Hui Du, Yong-Xiang Wei, Yan-Wen Qin

**Affiliations:** ^1^Beijing Key Laboratory of Upper Airway Dysfunction Related Cardiovascular Diseases, Beijing An Zhen Hospital, Capital Medical University, Beijing Institute of Heart, Lung and Blood Vessel Diseases, Beijing 100029, China; ^2^Key Laboratory of Remodeling-Related Cardiovascular Diseases, Ministry of Education, Beijing 100029, China; ^3^Otolaryngological Department of Beijing An Zhen Hospital, Capital Medical University, Beijing 100029, China; ^4^Department of Biochemistry and Molecular Biology, School of Basic Medicine, Tongji Medical College, Huazhong University of Science and Technology, Wuhan 430030, China

## Abstract

**Objectives:**

Obstructive sleep apnea (OSA) is a common disorder influenced by genetic and environmental factors. Mutations of *AT-hook DNA-binding motif containing 1 (AHDC1)* gene have been implicated which could cause rare syndromes presenting OSA. This study aims to investigate some rare mutations of *AHDC1* in Chinese Han individuals with OSA.

**Patients and Methods:**

Three hundred and seventy-five patients with OSA and one hundred and nine control individuals underwent polysomnography. A targeted sequencing experiment was taken in 100 patients with moderate-to-severe OSA, and genotyping was taken in 157 moderate-to-severe OSA and 100 control individuals. The effect of mutations was validated by the luciferase reporter assay.

**Results:**

One rare missense mutation (*AHDC1: p.G1484D*) and two mutations (c.-88C>T; c.-781C>G) in 5′-untranslated region (UTR) of *AHDC1* were identified. The rare mutation (c.-781C>G) in 5′-UTR that was identified in several patients presenting more severe clinical manifestations affects expression of *AHDC1. Conclusions*. Our results revealed three rare mutations of *AHDC1* in patients with OSA in Chinese Hanindividuals.

## 1. Introduction

Obstructive sleep apnea (OSA, OMIM 107650) is an increasingly common disorder, and it is characterized by repeated upper airway collapse during sleep that results in chronic intermittent hypoxia, hypercapnia, and sleep fragmentation [1, 2]. Affected individuals are at increased risks of cardiovascular disease, stroke, and other disorders [1]. However, the pathogenesis of OSA is unclear, so better understanding of the etiology of OSA is needed.

OSA is caused by multiple factors, most commonly obesity, muscle dysfunction, craniofacial abnormalities, and genetic predisposition [3–6]. Some Mendelian diseases also present OSA clinical features, such as auriculo-condylar syndrome [7], Costello syndrome [8], Xia–Gibbs syndrome [9], and Marfan syndrome [10]. OSA aggregates within families, having a first-degree relative with OSA increases risk of OSA by more than 1.5-fold [3]. Furthermore, approximately 35% to 40% of apnea-hypopnea index (AHI) which is the most common disease-defining metric for OSA may be explained by genetic factors [4, 11–14]. Simultaneously, from the pathological viewpoint, sympathetic nervous system activity, fat distribution, upper airway dilator muscle dysfunction, craniofacial development, heightened chemosensitivity, and a low arousal threshold may cause OSA [15, 16], whereas these pathological processes are also regulated by genes [17].

*AT-hook DNA-binding motif containing 1 (AHDC1)* gene is located on chromosome 1p36.11 and encodes 1603 amino acid protein AT‐hook DNA-binding motif-containing protein 1. And *AHDC1* is nearby *AT-rich interaction domain 1A (ARID1A)* gene, mutations in which cause autosomal-dominant Coffin-Siris syndrome, and its symptoms are maxillary hypoplasia, tongue cleft, poor alignment of teeth, etc. OSA could coexist with spinocerebellar ataxia type 1, which is a hereditary disease of the nervous system regulated by *AHDC1*. Abnormalities of *AHDC1* probably cause pharyngeal dilator muscle incoordination, thereby leading to airway collapse [9, 18, 19]. Moreover, mutations in *AHDC1* have been implicated to most likely cause Xia‐Gibbs syndrome. And Xia‐Gibbs syndrome presented OSA, language delay, and hypotonia. [9]. Two missense mutations in *AHDC1* were found to be associated with schizophrenia and oral developmental disorders that might lead to upper airway dysfunction and perhaps OSA [20, 21]. Nevertheless, if there were any rare mutations in *AHDC1* associated with OSA in Chinese Han is not clear.

The purpose of this study is to investigate rare mutations of *AHDC1* in Chinese Han individuals with OSA. In this study, we used targeted sequencing which is a cost-efficient tool [22] to discover rare mutations of *AHDC1* in unrelated Chinese Han individuals with OSA.

## 2. Patients and Methods

### 2.1. Patients

**Figure 1:**
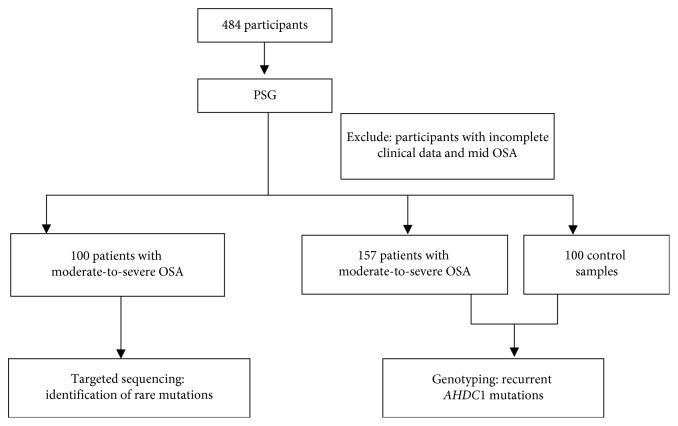
Study flow chart. PSG: polysomnography; OSA: obstructive sleep apnea.

Subjects for sequencing came from patients at the Otolaryngological Department of Beijing An Zhen Hospital from February 2017 to December 2017. All participants were subjects of self-reported snoring symptoms; each subject completed OSA screening and was scheduled for an overnight sleep study. The sleep study was conducted using a level II portable diagnostic device (SOMNOscreen; SOMNOmedics GmbH, Randersacker, Germany) approved by the US Food and Drug Administration. In total, 484 unrelated Chinese Han adults aged ≥18 years were recruited, including 375 patients with OSA and 109 control individuals. After excluding participants with incomplete clinical data and mild OSA, a targeted sequencing experiment was taken in 100 patients with moderate-to-severe OSA. To identify additional mutation carriers, we genotyped an expanded cohort of 157 moderate-to-severe OSA and 100 control individuals. The study flow chart is shown in Figure 1. Patients with OSA and control individuals were unrelated individuals diagnosed using the American Academy of Sleep Medicine guidelines [23]. All participants underwent a complete examination, and their medical history and basic clinical and biochemical variables were collected. No participants had lung, kidney, liver, or nervous system disease.

All participants completed an informed consent before enrollment. The study was approved by the Medical Ethics Committee of Beijing An Zhen Hospital (2017005), adhered to the Declaration of Helsinki, and was registered in the Chinese Clinical Trial Register (ChiCTR-ROC-17011027).

### 2.2. Phenotype Definitions

The diagnostic standard of OSA is defined as AHI more than 5 times per hour, and this standard is further subdivided into mild (5 < AHI ≤ 15 events/hour), moderate (15 < AHI ≤ 30 events/hour), and severe (AHI > 30 events/hour) [24]. The quantitative phenotypic outcomes were the AHI (defined by events associated with ≥3% desaturation), the lowest oxygen saturation (LSaO2) and mean oxygen saturation (MSaO2) across the sleep period. These quantitative phenotypic outcomes excluded intermittent waking episodes [25].

Covariates were obtained by questionnaires and direct measurement. Body mass index (BMI) was calculated using the standard BMI formula: body mass (in kilograms) divided by square of height (in meters). Hypertension is defined as having higher blood pressure than normal three times without antihypertensive drugs, i.e., systolic blood pressure ≥140 mmHg and/or diastolic blood pressure ≥90 mmHg. All blood samples were collected after participants had fasted overnight. Venous blood sample was obtained before 9 am. Clinical variables included total cholesterol (TC), triglyceride (TG), high-density lipoprotein cholesterol (HDL-C), low-density lipoprotein cholesterol (LDL-C), and fasting blood glucose (GLU). Serum TC, TG, HDL-C, LDL-C, GLU, and other routine serum biochemical parameters were measured using a biochemical analyzer (Hitachi-7600; Hitachi, Tokyo, Japan). Blood samples were stored in a freezer at −80°C. All measurements were obtained using blinded quality control specimens at the Department of the Biochemical Laboratory at Beijing An Zhen Hospital. All results were interpreted by a specialist.

### 2.3. Exome Sequencing Analysis

The genomic DNA was extracted from whole blood (details are described in the Methods section in the appendix). A custom-designed gene panel containing *AHDC1* (Supplementary [Supplementary-material supplementary-material-1]) was ordered from Life Technologies (Carlsbad, CA, USA) for targeted sequencing. The coverage of panel is 99.52% (Supplementary [Supplementary-material supplementary-material-1]). Details regarding primers and resequencing procedures are given in Supplementary [Supplementary-material supplementary-material-1] and the Methods section in the appendix.

The read alignments were filtered by the software into mapped BAM-files using the reference genomic sequence (hg19) of the target genes. Annotation of variants was performed using Ion Reporter Software (Version 4.4; Life Technologies, Darmstadt, Germany) for the variant call format files. The annotation included genomic and complementary DNA positions, genetic reference sequences, amino acid changes, and related information available from public databases, such as the 1000 Genomes Project; National Heart, Lung, and Blood Institute Grand Opportunity Exome Sequencing Project (ESP6500) (https://esp.gs.washington.edu/drupal/); Single Nucleotide Polymorphism Database (dbSNP147) (National Center for Biotechnology Information, http://www.ncbi.nlm.nih.gov/SNP/); Exome Aggregation Consortium (ExAC03) (http://exac.broadinstitute.org); ClinVar; Online Mendelian Inheritance in Man (OMIM); and Human Gene Mutation Database (HGMD). Sorting Intolerant From Tolerant (SIFT) (http://sift.jcvi.org/), PolyPhen-2 (http://genetics.bwh.harvard.edu/pph2/), MutationTaster (http://www.mutationtaster.org/), and Protein Variation Effect Analyzer (PROVEAN) (http://provean.jcvi.org/index.php) were used to indicate changes in protein structure and function. PathCards (http://pathcards.genecards.org/) was used to analyze the pathway. CLUSTALW (http://www.genome.jp/tools/clustalw/) was used to analyze the consistency of the amino acid sequences. Candidate pathogenic variants were confirmed using Sanger sequencing.

### 2.4. Functional Analysis

Bioinformatics analysis suggests that mutation (c.-781C>G) of *AHDC1* is located at 5′UTR of *AHDC1*. Two 1000-bp fragments containing human *AHDC1* 5′UTR-781C and -781G, respectively, were cloned into a pGL4.10[luc2] vector upstream of luciferase reporter (Promega Benelux BV, Leiden, The Netherlands), and the resultant plasmids (pGL4-C and pGL4-G) were transformed into 293T cells, respectively, to determine effects of the mutation by detecting fluorescence intensity according to the manufacturer's instructions.

### 2.5. Statistical Analysis

Continuous variables are expressed as mean ± standard deviation and categorical variables as numeral (percentage). Independent Student's *t*-tests for normal distributions and Wilcoxon rank sum tests for asymmetric distributions were used to analyze the differences in continuous variables. Chi-squared tests were used to analyze categorical variables between OSA and control group. Subsequently, independent Student's *t*-tests and Wilcoxon rank sum tests were used to analyze the differences in continuous variables, and Fisher's exact tests were used to analyze categorical variables between mutation and nonmutation groups. All probability values were two-sided, and *P* < 0.05 was considered significant. Statistical analyses were performed using SPSS 20.0 (IBM Corp., Armonk, NY, USA).

## 3. Results

### 3.1. Baseline Characteristics of Participants

The present study included 257 patients with OSA and 100 control individuals. Basal characteristics of these individuals are presented in Table 1. No significant differences were observed in age (*P*=0.055), TC level (*P*=0.569), or LDL-C level (*P*=0.400) between OSA and the control group. Compared with the control group, the patients had a higher BMI (27.04 ± 3.36 vs. 23.71 ± 3.09 kg/m^2^, <0.001), TG level (1.56 ± 1.04 vs. 1.24 ± 0.36 mmol/L, *P*=0.002), GLU level (3.46 ± 0.95 vs. 3.44 ± 0.62 mmol/L, *P*=0.002), HDL-C level (1.07 ± 0.23 vs. 1.00 ± 0.18 mmol/L, *P*=0.018), male proportion (84.82% vs. 70.00%, *P*=0.002), and prevalence of hypertension (22.96% vs. 13.00%, *P*=0.035). The OSA group exhibited severe degrees of AHI (32.17 ± 18.06 vs. 3.33 ± 1.14, *P* < 0.001), significantly lower MSaO2 (93.48 ± 2.28 vs. 95.00 ± 1.37) and LSaO2 (82.14 ± 9.89 vs. 90.00 ± 2.73, *P* < 0.001) in comparison with the control group.

### 3.2. Identification of Rare Mutations

In order to identify the mutation of *AHDC1* in Chinese OSA patients, we sequenced each of 100 moderate-to-severe OSA subjects' DNA samples using targeted sequencing technology. Among the 100 moderate-to-severe OSA patients, in total 57 variants were met the quality filtering criteria, 11 were common polymorphisms (i.e., minor allele frequency of ≥1%), whereas the remaining 46 were rare mutations (minor allele frequency of <1%). To prioritize potential deleterious variants, we focused on the identification of rare mutations (based on the 1000 Genomes, ESP6500, and ExAc databases). Four algorithms (SIFT, PolyPhen-2, MutationTaster, and PROVEAN) were used to predict whether the rare mutations in coding regions would change the protein structure and function. One heterozygous missense mutation (*AHDC1: c.G4451A, p.G1484D*) (Table 2) was identified in a 58-year-old male with AHI of 23.1, LSaO2 of 85%, and MSaO2 of 92%. We then analyzed mutations in noncoding regions; a mutation (*AHDC1: c.-88C>T*) in 5′-UTR of *AHDC1* was found in a 61-year-old male with AHI of 28.2, LSaO2 of 83%, and MSaO2 of 90%. Another rare variant in 5′-UTR of *AHDC1* (c.-781C>G) was identified in a 50-year-old male with AHI of 34.6, LSaO2 of 88%, and MSaO2 of 93% (Table 2).

To identify additional mutation carriers, genotyping was performed in a cohort of 157 moderate-to-severe OSA and 100 control individuals. Among 157 additional patients with OSA, the rare mutation in 5′-UTR of *AHDC1* (c.-781C>G) was found in a 61-year-old female with AHI of 68, LSaO2 of 69%, and MSaO2 of 88% (Table 3). In the control group, none of these three mutations was found. In summary, we found three rare mutations in two cohorts (*AHDC1: c.G4451A, c.-88C>T,* c.-781C>G) (Table 2), and the mutation in 5′-UTR of *AHDC1* (c.-781C>G) was found in two patients with OSA (Table 3). All left mutations were confirmed by Sanger sequencing (Supplementary [Supplementary-material supplementary-material-1]). The evolutionary conservation of the encoded amino acid residues (*AHDC1: c.G4451A*) was analyzed in nine different vertebrate species (Supplementary [Supplementary-material supplementary-material-1]). The residue that was mutated (*AHDC1: c.G4451A*) was found to be highly conserved.

### 3.3. Clinical Analysis in Patients with or without AHDC1 Mutations

To find whether OSA patients with *AHDC1* mutations had different clinical manifestations from the patients without mutations, we analyzed their clinical characteristics. No difference was found between these two groups in age (*P*=0.628), sex (*P*=0.578), and BMI (*P*=0.503) (Supplementary [Supplementary-material supplementary-material-1]). So distribution of mutations in *AHDC1* is not influenced by age, sex, or BMI in the present study.

Clinical manifestations of OSA patients with *AHDC1* mutations are shown in Table 3. Because HDL-C is less affected by drugs, we compared only HDL-C levels for biochemical indicators in patients with mutations. The patient with missense mutation (*AHDC1: c.G4451A*) was a moderate OSA patient (AHI = 23.1) with lower BMI (BMI = 17.3 kg/m^2^) and lower HDL-C (0.83 mmol/L), while he was a diabetic and coronary heart disease (CHD) patient. The mutation (c.-88C>T) was found in a fatter male (BMI = 25.3 kg/m^2^) who was a moderate OSA patient (AHI = 28.2) with normal HDL-C, TG, and fasting blood sugar level. Another mutation in 5′-UTR (c.-781C>G) was found in two severe OSA patients: the first severe OSA patient (AHI = 23.6) was a medium height male (BMI = 23.6 kg/m^2^) who was younger (50 years) than another three patients, and he was a hypertensive and CHD patient with lower HDL-C (0.84 mmol/L). The other severe OSA patient (AHI = 68,) was a fatter female (BMI = 34 kg/m^2^) with lower HDL-C (0.83 mmol/L), while she was a diabetic, hypertensive, and CHD patient; this female patient presented lower oxygen saturation (LSaO2 = 69%, MSaO2 = 88%).

### 3.4. Functional Analysis

**Figure 2:**
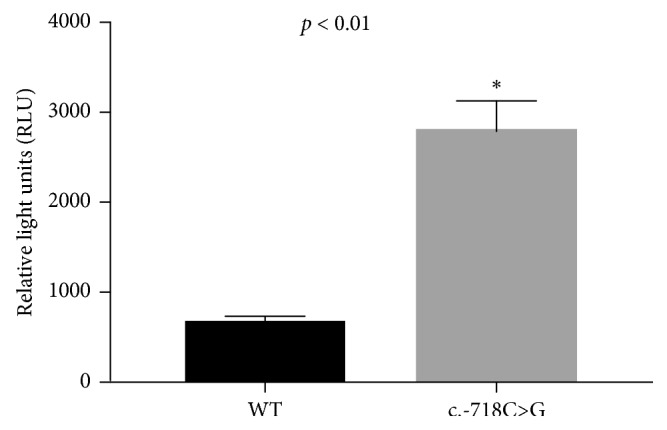
Luciferase activity. Compared with a negative control, luciferase activity increased with cotransfection of pGL4.10-G in both 293T cells.

Previous reports have shown that 5′ UTR may influence expression of genes. In this study, two patients with mutation in 5′UTR (c.-781C>G) presented higher AHI and lower oxygen saturation. So, the effect of mutation (c.-781C>G) in 5′UTR of *AHDC1* on its expression level was tested. The 293T cells were used for transfection of *AHDC1* 5′UTR-based reporter constructs (pGL4.10-C, with the C allele or pGL4.10-G, with the G allele). As shown in Figure 2, compared with a negative control, luciferase activity was found to be increased with cotransfection of pGL4.10-G in 293T cells (*P* < 0.01).

## 4. Discussion

In this study, we identified one rare missense mutation (*AHDC1: p.G1484D*) and two rare mutations (c.-88C>T  and c.-781C>G) in 5′-UTR of *AHDC1* in OSA patients. Functional analysis and luciferase reporter assay suggested that the mutation (c.-781C>G) in 5′-UTR played an important role in *AHDC1*.

**Figure 3:**
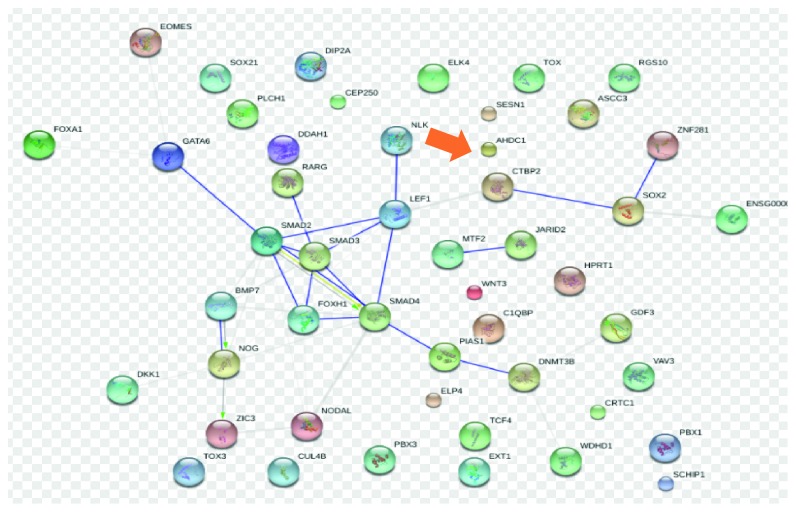
STRING interaction network for Mesodermal Commitment Pathway (SuperPath).

Previous studies have found that the carriers of *AHDC1* mutation presented pharyngeal dilator muscle incoordination and oral developmental disorders [26]. In the present study, MalaCards database suggests an important gene associated with sleep apnea is *AHDC1*, and among its related pathways/superpathways are signaling by GPCR and respiratory electron transport, ATP synthesis by chemiosmotic coupling, and heat production by uncoupling proteins. *AHDC1* is also associated with *Mesodermal Commitment Pathway* (Figure 3) that may influence development of respiratory-related muscles and bones leading to OSA. These evidences suggest that *AHDC1* might play an important role in OSA.

The missense mutation (*AHDC1: c.G4451A*) was found in a thinner moderate OSA patient. This suggests that OSA may occur in the subject with *AHDC1* mutations, even if his or her BMI is small. The mutation in 5′UTR (c.-88C>T) was found in a moderate OSA patient with normal biochemical variables, and he did not take any drugs. This suggests one interesting fact that not all OSA patients might suffer from other diagnosed diseases; every patient has his or her own characteristics, so individualized diagnosis and treatment are necessary. On the other hand, this mutation (c.-88C>T) may not cause changes in pathways related to other diseases, so we have not performed further functional analysis of this mutation. Moreover, another interesting rare mutation in 5′UTR (c.-781C>G) was found in two patients with higher AHI, lower oxygen saturation, and lower HDL-C. Analysis of potential transcriptional binding sites in the proximate promoter region (1-kb of upstream region of the start site of *AHDC1*) revealed that the c.-781C>G-containing sequence is the binding consensus motif of the transcription factor SREBP-2 (Supplementary [Supplementary-material supplementary-material-1]). Mutations of *SREBP-2* were found to be related to hypercholesterolemia and perhaps OSA [27]. And luciferase reporter assay suggests the mutation (c.-781C>G) in 5′-UTR of *AHDC1* affects expression of gene. All above suggest that *AHDC1* may be related to the occurrence of OSA and 5′UTR plays a significant role in *AHDC1.*

Notably, a recent whole-exome sequencing analysis revealed three different *de novo* truncating mutations in *AHDC1* in four patients with OSA, language delay, and hypotonia [9]. Two missense mutations in *AHDC1* were found to be associated with schizophrenia and oral developmental disorders that may lead to upper airway dysfunction and perhaps OSA [20, 21]. Previous founded variants are not only in the coding exon [9] but also in 5′ untranslated region [21]. A *de novo* balanced translocation with a breakpoint in *AHDC1* intron 1 that disrupted the 5′UTR of *AHDC1* has been identified in a 5-year-old male patient with developmental delay and intellectual disability [21]. The disruption lead to *AHDC1* expression reduced to 50% of wild-type level in lymphoblastoid cells [21]. In the present study, rare mutation (c.-781C>G) in 5′UTR of *AHDC1* was found in severe OSA patients, and this rare mutation could affect expression of *AHDC1.* Therefore, we assumed that the mutations in *AHDC1* may affect the expression of *AHDC1* and lead to sleep apnea. The exact mechanism of this phenomenon needs to be investigated.

This study has some limitations. The participants may not be entirely representative of the general Han Chinese population. Potential false-positive results may still be possible. Prospective cohort studies are needed to confirm the variants in our study. We sequenced limited genes and limited sequencing regions that were most likely to harbor functional variation. Some of the patients and controls with CHD were taking drugs. The results of TC, LDL-C, and TG comparison between two groups may be different from those of other studies. Finally, the exact mechanism of these variants is not fully understood and requires further functional studies.

## 5. Conclusions

In summary, we identified multiple novel variants of *AHDC1* in patients with OSA. Our findings increase the mutation spectrum of associated genes for OSA, a quite complex disease. Gene analysis is helpful for individualized typing of patients with OSA and might contribute to personalized diagnosis and treatment in the future.

## Figures and Tables

**Table 1:** Baseline characteristics of participants.

Measure	OSA group	Control group	*P*
Number of subjects	257	100	—
Age (years)	58.97 ± 9.41	58.11 ± 10.92	0.055
Sex (% male)	84.82	70.00	0.002^*∗*^
BMI (kg/m^2^)	27.04 ± 3.36^#^	23.71 ± 3.09	<0.001^*∗*^
TC (mmol/L)	4.13 ± 1.00	3.99 ± 1.02	0.569
TG (mmol/L)	1.56 ± 1.04^#^	1.24 ± 0.36	0.002^*∗*^
HDL-C (mmol/L)	1.07 ± 0.23	1.00 ± 0.18	0.018^*∗*^
LDL-C (mmol/L)	2.47 ± 0.87^#^	2.43 ± 0.83	0.400
GLU (mmol/L)	3.46 ± 0.95^#^	3.44 ± 0.62	0.002^*∗*^
Hypertension (%)	22.96	13.00	0.035^*∗*^
AHI	32.17 ± 18.06^#^	3.33 ± 1.14	<0.001^*∗*^
MSaO2 (%)	93.48 ± 2.28^#^	95.00 ± 1.37 ^#^	0.002^*∗*^
LSaO2 (%)	82.14 ± 9.89^#^	90.00 ± 2.73	<0.001^*∗*^

Data are expressed as mean ± standard deviation or *n* (%). Differences between groups were analyzed by the independent Student's *t*-test, chi-squared test, or Wilcoxon test. ^#^Data were asymmetrically distributed. ^*∗*^*P* < 0.05. BMI: body mass index; TC: total cholesterol; TG: triglyceride; HDL-C: high-density lipoprotein cholesterol; LDL-C: low-density lipoprotein cholesterol; GLU: fasting blood glucose; AHI: apnea-hypopnea index; MSaO2: mean oxygen saturation; LSaO_2_: lowest oxygen saturation.

**Table 2:** Probably damaging rare mutations.

Locus	Gene	Base change	AA change	SIFT	PPT2	MutationTaster	PRO VEAN
Chr1：27874176	AHDC1	c.G4451A	p.G1484D	D	D	D	N
Chr1：27879416	AHDC1	c.-88C>T	UTR5	—	—	—	—
Chr1：27879407	AHDC1	c.-781C>G	UTR5	—	—	—	—

AA: amino acid; SIFT (D: deleterious, T: tolerated); PolyPhen-2 (i.e., PPT2) (D: probably damaging, P: possibly damaging, B: benign); MutationTaster and PROVEAN (D: disease-causing, N: polymorphism).

**Table 3:** Clinical features of patients with rare mutations.

ID	Age (yr)	Variation	Sex	BMI (kg/m^2^)	AHI	LSaO2 (%)	MSaO2 (%)	TC (mmol/L)	TG (mmol/L)	HDL-C (mmol/L)	LDL-C (mmol/L)	GLU (mmol/L)	Hypertension	CHD
1	58	p.G1484D	Male	17.3	23.1	85.0	92.0	3.20	2.63	0.83	1.74	17.56	No	Yes
2	61	c.-88C>T	Male	25.3	28.2	83.0	90.0	4.02	1.11	1.17	2.58	2.94	No	No
3	50	c.-781C>G	Male	23.6	34.6	88.0	93.0	3.95	0.86	0.84	2.76	4.45	No	Yes
4	61	c.-781C>G	Female	34.0	68.0	69.0	88.0	2.23	2.72	0.83	0.79	12.96	Yes	Yes

yr: years; BMI: body mass index; AHI: apnea-hypopnea index; LSaO2: lowest oxygen saturation; TC: total cholesterol; TG: triglyceride; HDL-C: high-density lipoprotein cholesterol; LDL-C: low-density lipoprotein cholesterol; GLU: fasting blood glucose; CHD: coronary heart disease.

## Data Availability

The data used to support the findings of this study are available from the corresponding author upon request.

## Supplementary Materials

Supplementary Materials Supplementary Table 1: detailed information of targeted sequencing panel. Supplementary Table 2: AmpliSeqT amplicons and coverage details of obstructive sleep apnea-targeted sequencing assay. Supplementary Table 3: primers of panel. Supplementary Table 4: characteristics between mutation and nonmutation groups. Supplementary Figure 1: Sanger sequencing chromatograms of variants in AHDC1. Supplementary Figure 2: analysis of potential transcriptional binding sites in the proximate promoter region. Supplementary Figure 3: analysis of sequence conservation in nine species of missense mutation identified in AHDC1.

## References

[1] Cade B. E., Chen H., Stilp A. M. (2016). Genetic associations with obstructive sleep apnea traits in Hispanic/Latino Americans. *American Journal of Respiratory and Critical Care Medicine*.

[2] Peppard P. E., Young T., Barnet J. H., Palta M., Hagen E. W., Hla K. M. (2013). Increased prevalence of sleep-disordered breathing in adults. *American Journal of Epidemiology*.

[3] Strohl K. P., Saunders N. A., Feldman N. T., Hallett M. (1978). Obstructive sleep apnea in family members. *New England Journal of Medicine*.

[4] Palmer L. J., Buxbaum S. G., Larkin E. (2003). A whole-genome scan for obstructive sleep apnea and obesity. *The American Journal of Human Genetics*.

[5] Kripke D. F., Kline L. E., Nievergelt C. M. (2015). Genetic variants associated with sleep disorders. *Sleep Medicine*.

[6] Varvarigou V., Dahabreh I. J., Malhotra A., Kales S. N. (2011). A review of genetic association studies of obstructive sleep apnea: field synopsis and meta-analysis. *Sleep*.

[7] Gordon C. T., Vuillot A., Marlin S. (2013). Heterogeneity of mutational mechanisms and modes of inheritance in auriculocondylar syndrome. *Journal of Medical Genetics*.

[8] Marca G. D., Vasta I., Scarano E. (2006). Obstructive sleep apnea in Costello syndrome. *American Journal of Medical Genetics Part A*.

[9] Xia F., Bainbridge M. N., Tan T. Y. (2014). De novo truncating mutations in AHDC1 in individuals with syndromic expressive language delay, hypotonia, and sleep apnea. *The American Journal of Human Genetics*.

[10] Mo L., He Q., Wang Y., Dong B., He J. (2014). High prevalence of obstructive sleep apnea in Marfan’s syndrome. *Chinese Medical Journal*.

[11] Palmer L. J., Buxbaum S. G., Larkin E. K. (2004). Whole genome scan for obstructive sleep apnea and obesity in African-American families. *American Journal of Respiratory and Critical Care Medicine*.

[12] Carmelli D., Colrain I. M., Swan G. E., Bliwise D. L. (2004). Genetic and environmental influences in sleep-disordered breathing in older male twins. *Sleep*.

[13] Zhang D., Xiao Y., Luo J. (2014). Genetics of obstructive sleep apnea/hypopnea syndrome. *Chinese Medical Journal*.

[14] Redline S., Tishler P. V., Tosteson T. D. (1995). The familial aggregation of obstructive sleep apnea. *American Journal of Respiratory and Critical Care Medicine*.

[15] Campana L., Eckert D. J., Patel S. R., Malhotra A. (2010). Pathophysiology & genetics of obstructive sleep apnoea. *The Indian Journal of Medical Research*.

[16] Jordan A. S., McSharry D. G., Malhotra A. (2014). Adult obstructive sleep apnoea. *The Lancet*.

[17] Redline S., Tishler P. V. (2000). The genetics of sleep apnea. *Sleep Medicine Reviews*.

[18] Lim J., Hao T., Shaw C. (2006). A protein-protein interaction network for human inherited ataxias and disorders of Purkinje cell degeneration. *Cell*.

[19] Dang D., Cunnington D. (2010). Excessive daytime somnolence in spinocerebellar ataxia type 1. *Journal of the Neurological Sciences*.

[20] Guipponi M., Santoni F. A., Setola V. (2014). Exome sequencing in 53 sporadic cases of schizophrenia identifies 18 putative candidate genes. *PLoS One*.

[21] Quintero-Rivera F., Xi Q. J., Keppler-Noreuil K. M. (2015). MATR3 disruption in human and mouse associated with bicuspid aortic valve, aortic coarctation and patent ductus arteriosus. *Human Molecular Genetics*.

[22] Mardis E. R. (2008). The impact of next-generation sequencing technology on genetics. *Trends in Genetics*.

[23] Berry R. B., Budhiraja R., Gottlieb D. J. (2012). Rules for scoring respiratory events in sleep: update of the 2007 AASM manual for the scoring of sleep and associated events. Deliberations of the sleep apnea definitions task force of the American Academy of Sleep Medicine. *Journal of Clinical Sleep Medicine*.

[24] Foster G. D., Borradaile K. E., Sanders M. H. (2009). A randomized study on the effect of weight loss on obstructive sleep apnea among obese patients with type 2 diabetes: the Sleep AHEAD study. *Archives of Internal Medicine*.

[25] Cheung Y. Y., Tai B. C., Loo G. (2017). Screening for obstructive sleep apnea in the assessment of coronary risk. *The American Journal of Cardiology*.

[26] Javaheri S., Barbe F., Campos-Rodriguez F. (2017). Sleep apnea: types, mechanisms, and clinical cardiovascular consequences. *Journal of the American College of Cardiology*.

[27] Li J., Grigoryev D. N., Ye S. Q. (2005). Chronic intermittent hypoxia upregulates genes of lipid biosynthesis in obese mice. *Journal of Applied Physiology*.

