# Supplementary material for: Rare Mutations in AHDC1 in Patients with Obstructive Sleep Apnea
Source: Biomed Res Int. 2019 Oct 13;2019:5907361. doi: 10.1155/2019/5907361 (PMC6815587; doi:10.1155/2019/5907361)
Supplement: Supplementary Materials — Supplementary Table 1: detailed information of targeted sequencing panel. Supplementary Table 2: AmpliSeqT amplicons and coverage details of obstructive sleep apnea-targeted sequencing assay. Supplementary Table 3: primers of panel. Supplementary Table 4: characteristics between mutation and nonmutation groups. Supplementary Figure 1: Sanger sequencing chromatograms of variants in AHDC1. Supplementary Figure 2: analysis of potential transcriptional binding sites in the proximate promoter region. Supplementary Figure 3: analysis of sequence conservation in nine species of missense mutation identified in AHDC1. [file 5907361.f1.pdf]

Rare mutations in AHDC1 in Patients with Obstructive Sleep Apnea

Appendix

DNA template preparation and amplification

DNA was extracted from 200 μl of blood according to the protocol provided in the QIAamp DNA Mini Kit (Qiagen, Hilden, Germany). The multiplex polymerase chain reaction (PCR) amplification strategy for DNA sequences was accomplished online (Ion Ampliseq™ Designer; http://www.ampliseq.com) to amplify the target region (for primer sequences, **Supplementary Table 3**). Then the maximum coverage design was chosen. A total of 10 ng of DNA per sample was used for enrichment by multiplex PCR, each DNA pool was amplified with the Ion Ampliseq™ Library Kit; the manufacturer's protocols were followed (Life Technologies, Darmstadt, Germany).

After each pool undergone 17 PCR cycles, the PCR primers were removed with FuPa Reagent (Life Technologies), next the amplicons were ligated to the sequencing adaptors with barcodes (Ion Xpress™ Barcode Adapters Kit; Life Technologies). After purification with AMPure XP beads (Beckman Coulter, Krefeld, Germany), the barcoded libraries were quantified with a Qubit® 2.0 Fluorimeter (Life Technologies), then the DNA was concentrated to 20 pmol/L using the Ion Library Equalizer™ Kit (Life Technologies). The IT OneTouch template kit on an IT OneTouch system (Life

Technologies) were used to clonally amplify the library DNA onto the Ion Sphere Particles (Life Technologies).

Sequencing

Ion 318 Chip was used to carry copies of the DNA fragment. Sequencing was performed using the sequencing kit (Ion PGM Sequencing Kit; Life Technologies) according to the manufacturer’s instructions with the 400-bp single-end run configuration.

Supplementary Table 1 Detailed information of targeted sequencing panel

GeneName	Locus	OMIM number	Protein	Coverage	Function	Ref
**AHDC1**	1p36.11-p35.3	615829	A protein containing two AT-hooks, which likely function in DNA binding	0.995	Syndromic Expressive Language Delay, Hypotonia, and Sleep Apnea	[1]

Supplementary Table 2 AmpliSeq™ amplicons and coverage details of obstructive sleep apnea-targeted sequencing assay

Target_ID	Gene	Chr	Chr_Start	Chr_End	Total_Bases	Covered_Bases	Missed_Bases	Coverage
1919052	AHDC1	chr1	27860750	27861432	682	651	31	0.955
1919049	AHDC1	chr1	27873766	27878705	4939	4939	0	1
1919055	AHDC1	chr1	27879397	27879445	48	48	0	1
1919050	AHDC1	chr1	27879579	27879806	227	227	0	1
1919054	AHDC1	chr1	27884810	27885046	236	236	0	1
1919053	AHDC1	chr1	27885211	27885399	188	188	0	1
1919051	AHDC1	chr1	27929902	27930148	246	246	0	1

Supplementary Table 3 Primers of panel

Ion_AmpliSeq_Fwd_Primer*	Ion_AmpliSeq_Rev_Primer*	Name	Amplicon_Start	Amplicon_Stop
TACGGATGCTGTTGGCCAAA	GATTGACATCATGAAGGACCTGACA	AHDC1	27878092	27878255
GCCAGGACGTCTGAGTACTC	CGGAACGTGGTAGTGGTCTT	AHDC1	27876777	27877045
CCAGAGGATCAGCAAGAGGTTG	CAGACAGCACTGATTACTCAGAACTT	AHDC1	27877705	27877935
GCCTTGCCCTTAAATCCTGCAT	GCCCACTCCTTAGTGACTTGAC	AHDC1	27873652	27874008
GTACCCACCTTGCCTTCATAGG	GAGCTTGATGGCAAGCATTTCC	AHDC1	27874173	27874507
GTGGAAGCCTTGGCCAAAGGT	CCTCAGGCTACCCATCCAAAC	AHDC1	27874535	27874857
AGAGGACACTCTGGTTCCAGTT	TCAGAGGCCTTTAGTCAGCTCTA	AHDC1	27874954	27875266
GTGCCCTTGGGTACCATGTAG	GCCGGACAAAGCGTATTCG	AHDC1	27875406	27875701
GTCTGAAGCGGCTGGAGTTA	CCTCTTCACCGGCTACTTTCG	AHDC1	27875816	27876125
GCTCTGGGAACAGAGTCCCAT	CAAAGCAGGCGGTTTTGGT	AHDC1	27876367	27876617
CTTTGCGGGTAGAGACCACT	CCTTGCGCCTGGACTTGT	AHDC1	27877231	27877542
CGCCTCGGCTCACATTCT	CCCTCCCAGGTTTGCTATTGG	AHDC1	27930066	27930247
GCGTGGGTTCTCGGAGAAG	TAAGGGACCTCCTGCTCTTCC	AHDC1	27878441	27878731
GTGGGCAACAAGACGGGCAATA	CCCTACCTCACAGGATTGGTATGAA	AHDC1	27879443	27879814
CAGATCTCAGGCAGAAGGGAAAA	CACTCTTTTGGGTTTTCTCTTCTGTTG	AHDC1	27860610	27860894
GGATATTGTTCATTTTTGTCAAAC GACCT	GCACAAAACCAGCAATTGTAAATACTT TTT	AHDC1	27861043	27861259
TGATTCTGTAAAGAAGCTCTTTTT GACCT	GCTTACCCTGTGTCCATGCTTA	AHDC1	27885085	27885445

GCAGGCTTCATGTGTCAGTTCA	GCCTTGATGACCTGAGAGAACTG	AHDC1	27884619	27884975
CCTGGCCTGTAAAGGGCTTA	AGGACGAGCAGCAAGCTATG	AHDC1	27875656	27875858
GCATAAAGCCTGGGTACCTGTAG	AAGGGCACAGCCTATTGGTAC	AHDC1	27873916	27874243
CTCTTCCTTGAAGCCCAGTGTA	ACTTCATAGGACCCTACTCCATGAAC	AHDC1	27874394	27874602
TCAGGACTGTCCTGGAACAGT	ACTGGAACGAGGCATCATCTG	AHDC1	27874695	27875007
CGCTGACGTGGCAGTCAA	AAGCAAGGCCTCCTTCTTCAG	AHDC1	27875215	27875531
AGATCGGAGGAGTCATCCGAAT	CTGTGACTGGGAAGCCAAAG	AHDC1	27876072	27876429
CTCAAAGAAGTCACTGAAGGAGCAT	ATGGCCATGCCAGAGGTAAAA	AHDC1	27876539	27876892
GCTTACGTTTGAGAATAATGGGCATCT	GTCCCAGAGTTGAAGCCGGAAT	AHDC1	27876988	27877285
CGACACAGGATCTTTGGCCTA	CTGGACCCGCAGGCACTAGA	AHDC1	27877443	27877780
GAGGCCAGCTCACTAAGGATG	GCGACCTACAGTACAGCTTCTTC	AHDC1	27877878	27878145
TGTACACCACTGTGCTGCAG	ACCTCCGGGAACCCAAGTA	AHDC1	27878196	27878565
CTGTCCAGGCATCGTTTCATTT	CAGTCCTTGTCCAAAGTAGGTACTTTATAA	AHDC1	27879763	27879925
CGTGCGCTGCACAAAAGAG	CCGTGTCCAGCGGAGTTAG	AHDC1	27929834	27930108
GTGTGTCCTGCCAACAGAAAAC	CCCTGCAAGTATGCACTGAGAA	AHDC1	27860835	27861103
GGCCCAGTTTCACTTATTGACAG	GGAATTCAGATCTATTGCCCGTCTT	AHDC1	27879204	27879476
AAGTATTTACAATTGCTGGTTTTGTGCA	CCTGCCTCTTCTCTTGTGTTTCTT	AHDC1	27861233	27861456
TGAGTCCTGGTCATTGTCACAAC	GGTCAAAAAGAGCTTCTTTACAGAATC	AHDC1	27884917	27885112

	AAA			

Primers were designed using the Web based Ion AmpliSeq Designer software (https://www.ampliseq.com). This tool generates different primer design solutions, which differ for specificity, sample type, and application. After comparison of the primer design options, the design providing the maximum coverage was chosen.

Ion_AmpliSeq_Fwd_Primer*: Forward primer sequence.

Ion_AmpliSeq_Rev_Primer*: Reward primer sequence

Supplementary Table 4 Characteristics between mutation and non-mutation group

Measure	Mutation group	Non-mutation group	P
**Number of subjects**	4	353	-
**Age (years)**	57.5 ± 5.20	54.68 ±11.55#	0.628
**Sex(% male)**	75.00	80.74	0.578
**BMI (kg/m2)**	25.07 ± 6.89	26.33 ± 3.80#	0.503

Data are expressed as mean ± standard deviation or n (%). The differences between groups were analyzed by the independent Student's t-test, Wilcoxon test or Fisher's exact tests. #Data were asymmetrically distributed. *P<0.05

BMI: body mass index,

Supplementary Figure 1 Sanger sequencing chromatograms of variants in *AHDC1*.

1. AHDC1 (c.-781C>G). chr1: 27879407 G C

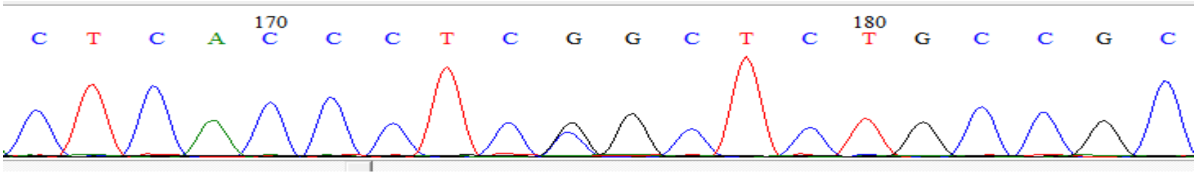

2. AHDC1 (c.-88C>T). chr1: 27879416 **C T**

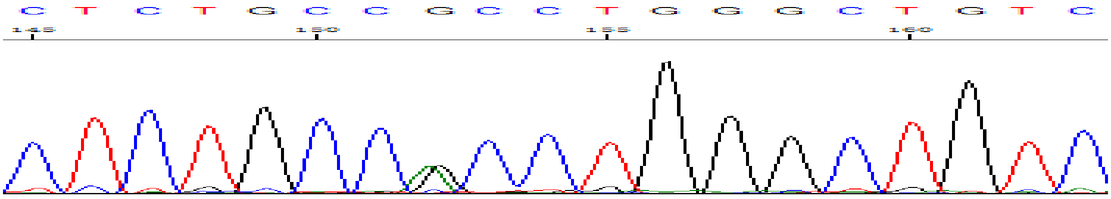

3. AHDC1: p.G1484D chr1: 27874176 C T

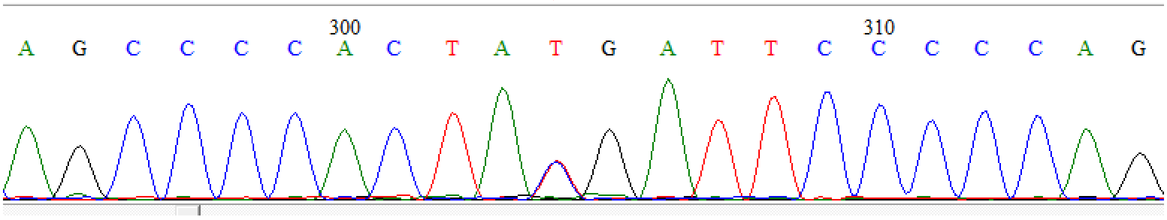

Supplementary Figure 2 Analysis of potential transcriptional binding sites in the proximate promoter region

>NG_034158.1:55380-56379 Homo sapiens AT-hook DNA binding motif containing 1 (AHDC1), RefSeqGene on chromosome 1 (1000-bp) the variant is at nt 55598

GACTGGCAGAGCCCCCAGGCACGGGGGCCCTCTCTCTTCCAGGTCAGCCGGGTGTCT
GCGCTGGACAGAGCGCTGAGGGAGCCGGGGGTGGTACCTGGAGGAGGCTGGGCCGGT
GGGCAGGGGTTGAGCTGTGGCCACCAGACCATTTGGGGAATTCAGATCTATTGCCCG
TCTTGTTGCCCACAGAGATCTCCAGTGTGACAGCCCAGGCGGCAGAGCCGAGGGTGA
GTGGTGGGGGTTCTCCTGGAGCCCTTTCGGGGTAAGAAGGAGGGAGGCCGTGCCCTT
GTTAGAACTTCCTGGCCTTCTCCTTAAGTTTGATCACCCTGCTGATGTGCTTCCCTC
TCAAAGGCAGGGGTCCTGTTCTTCCTGAGTGCATAGCCAGATTCCCAAAGGGGATGA
GCTGTCAATAAGTGAAACTGGGCCGGGCATGGTGGCTCACGCCTGTAATCCCAGTAT
TTTGGGAGGCTGAGGCAAGTGGATCATCTGAGGTCAGGAGCTCCAGACCAGCCTGGC
CAACTTGGTGAAACCCCGTCTCTACTAAAATACAAAAATTAGCCAGGTGTGGTGGCG
CATGCCTGTAATCCCAGGTGCTTGGGAGGCTGACACCTGAGAATCGCTTGCACTAGG
AGGCGGAGGTTGCAGTGAGCTGAGATCACGCTGCTTCACTCTAGCCTGGGCGACAGA
GTGAGACTCTCTTCCAAAAAAAAAAAAAAAAGTGAAACTGGGCCCATGAGGGGCTCA
CTATACACATGGTGGTTTGAGAAAGGCTGGGAGTCAGCAAGGGGATGGGGCTTGGGG
TGGGGAGGGGAGCTTGGTAAGGAGATGAGACCTCAGGAGGGAGGGGTCCAGGAGCTC
ATCAAGGATATGGGAAGCTGATGAGGATGTAGGTGTTCAGTAAGGGACCTCCTGCTC
TTCCTGCCTGGCAGGTCCTGGTCCCGGCTTCTCGCACCCTCATGTCAGCCCCGGCCC
CGCCCGGCGGCCTGCGGAGGACAAGGTCAGG

219 (-)	ccgagGGTGAg

The variant is at -219 or at +782

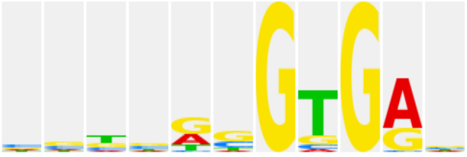

Factor name: SREBP-2
Matrix: V$SREBP2_Q6_01
From: 219 (-)
To: 229
Matrix score: 0.984
Core score: 1.000
Sequence: ccgagGGTGAg (click to copy)

Supplementary Figure 3 Analysis of sequence conservation in nine species of missense mutation identified in *AHDC1*.

AHDC1: p.G1484D

p.G1484D

↓

EGKVGTGLL	Human
EGKVGTGLL	Chimpanzee
- -- -- ----	Rhesus
EGKVGTGLL	Dog
EGKVGSGLL	Mouse
EGKVGSGLL	Rat
-- - -- ----	Fugu
-- - -- ----	Chicken
-- - -- ----	Zebrafish

All variants were subjected to the sequence conservation analysis in nine species, including human (*Homo sapiens*), chimp (*Pan troglodytes*), rhesus (*Macaca mulatta*), dog (*Canis familiaris*), mouse (*Mus musculus*), rat (*Rattus norvegicus*), torafugu (*Takifugu rubripes*), chicken (*Gallus gallus*), and zebrafsh (*Danio rerio*). The results showed that AHDC1:p.G1484D was evolutionarily conserved in vertebrates.

References

[1] F. Xia, M. N. Bainbridge, T. Y. Tan et al., "De novo truncating mutations in AHDC1 in individuals with syndromic expressive language delay, hypotonia, and sleep apnea," *American journal of human genetics,* vol. 94, no. 5, pp. 784-789, 2014.
